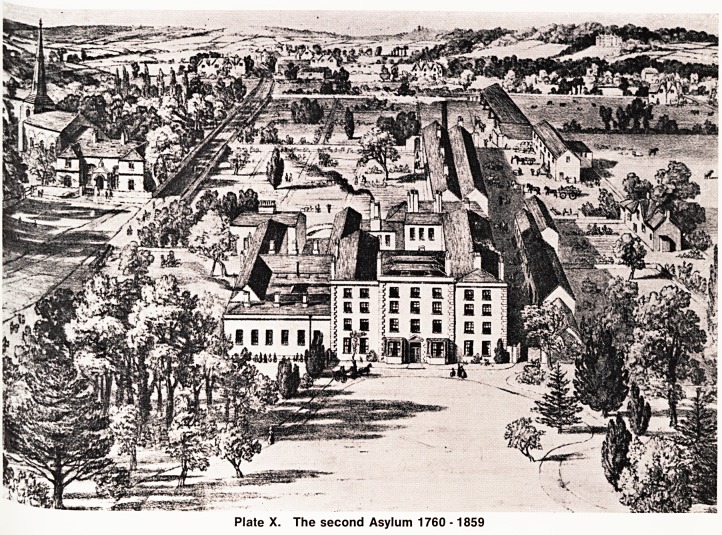# The Old Private Lunatic Asylum at Fishponds

**Published:** 1970-04

**Authors:** H. Temple Phillips

**Affiliations:** Lecturer in Public Health, University of Bristol; Senior Principal Medical Officer, City and County of Bristol


### The Old Private Lunatic Asylum at Fishponds by

**H. Temple Phillips, M.D., D.P.H., D.C.H., D.I.H.**

Lecturer in Public Health, University of Bristol; Senior Principal Medical Officer, City and County of Bristol.

Fishponds has long been associated with the care of the mentally ill. The Bristol Borough Lunatic Asylum (now Glenside Hospital) was established there in 1861, but it may not be generally known that there was a private lunatic asylum in Fishponds as early at 1740.

In 1738, on the death of his father, Dr. Joseph Mason (Plate VIII) succeeded to the management of a small mental establishment at Wickwar. A year or two later he moved to Fishponds, and the following advertisement appeared in the Gloucester Journal in November, 1740:—"This is to give notice that Dr. Joseph Mason of Wickwar, in the County of Gloucester, undertakes to cure Hypochondriacs, Mad and Distracted People, with great success. He is lately removed to a House more agreeable to that Business called by the name of Turvey's Corner, in the parish of Stapleton, two Miles from Bristol, in the same County: and will undertake, with the Blessing of God, to cure persons so disordered. No Cure, No Pay. Boarding excepted. His constant Method has been to keep them with good Usage, and wholesome Food, having now several Persons of Distinction under his Care. N.B. He will undertake to cure the King's Evil. No Cure, No Pay, if the Bones be not foul."

I have not been able to find any other reference to "Turvey's Corner", but we do know, from the Kingswood Enclosure Commissioners' Book of 1779, that Joseph Mason at one time rented the building which was later the Stapleton Poor House (Robinson and Hudleston 1938). We also know that the site of the old Stapleton Poor House is now occupied by Hygienex Industries Ltd. (formerly the Hygienic Straw Company) of College Road, Fishponds. This site corresponds exactly with "Mason's Madhouse" on an eighteenth century map of Bristol by Benjamin Donn. Part of the original building is still standing, being used as offices by Hygienex Industries Ltd. (Plate IX). This part of the building was almost certainly Dr. Mason's residence.

Little is known of the original "Mason's Madhouse", but Evans (1824) states that in 1746 the Town Clerk of Bristol (Sir William Cann), his deputy, and their clerk were all three insane. The Town Clerk cut his own throat, and the other two were sent to "the receptacle at the Fishponds".

In 1760 Mason removed his asylum to new premises about a quarter of a mile away—an imposing four-storeyed house in the style of the period. None of this second asylum remains, but its site corresponds to an area bounded by Manor Road, Oldbury Court Road, College Avenue, and College Road. The house contained its own private chapel and about 25 bedrooms for staff and patients.

Dr. Joseph Mason was a leading member of the Broadmead Baptist Chapel. He died in 1779 and his will, a very lengthy one, throws some light on his character. He was clearly a highly religious man, and he desired, among other things, "that the religious worship be constantly attended to night and morning in my now dwellinghouse in the same way it is now". His obituary notice referred to him as "universally known for his uncommon benevolence and many social virtues by all who had the happiness of being acquainted with him".

After Joseph Mason's death, the Fishponds Private Lunatic Asylum was managed for some years by his two married daughters, Elizabeth Cox and Sarah Carpenter, but in 1788, his grandson, Dr. Joseph Mason Cox, assumed control. Mason Cox had obtained his M.D. at Leyden, having previously studied at London, Edinburgh, and Paris. The title of his thesis (dated 1787) was "Quaedam de Mania".

He is best remembered for his textbook "Practical Observations on Insanity", which he published in 1804. A second edition came out in 1806 and a third in 1813. Daniel Hack Tuke (1882) called it "the best medical treatise of the day on insanity", but a contemporary review in the Edinburgh Medical and Surgical Journal (1805) was less laudatory, referring to Cox as "a lamentable instance . . . of the impossibility of writing anything satisfactory on this subject", and deploring that he should have been induced to hazard thereby his reputation. All three editions are to be found in the Bristol University Medical Library. One of Mason Cox's favourite treatments was what he called "swinging . . . effected by suspending a common Windsor chair to a hook in the ceiling . . . the patient being secured in a strait waistcoat and . . . prevented from falling out of the chair by a broad leather strap. . . . The patient thus

**Plate VIII. Dr. Joseph Mason 1711 - 1779**

secured and suspended a few inches from the ground, the motion may be communicated by an attendant turning him round." He was also an advocate of the sudden application of, or immersion in, cold water (le bain de surprise) — a treatment perhaps no more empirical than some of those employed today.

Mason Cox built himself a residence at Overn Hill (Downend), where, according to Richard Smith Junior (c. 1820), he employed all his leisure on the gardens, planting almost everything with his own hands. He was also passionately fond of music, and for many years belonged to a quartet party. He died in 1818, having been in poor health for some years. An autopsy performed by Nathaniel Smith and Richard Smith Junior showed that "the heart was enlarged to at least double the usual size and ossified to a considerable extent." He had previously consulted many of his medical colleagues, most of whom diagnosed a disease of the stomach.

For the next twenty-nine years the asylum was managed by Dr. George Gwinnett Bompas, Mason Cox's second cousin. The accompanying picture (Plate X) probably belongs to the latter part of this period, and shows Joseph Mason's original house, together with a number of additions made at various times by both Mason Cox and George Gwinnett Bompas. To the left of the picture, St. Mary's Church, Fishponds, and the old Free School (Hannah More's birthplace) are easily recognisable.

Surprisingly little information survives as to the asylum in the time of George Gwinnett Bompas. However, a pathetic fragment has recently found its way into the Bristol City Archives, in the form of a letter, written by a patient Maria Acland in 1838, and "dropped surreptitiously in the road outside." In it she paints a black picture of conditions in the asylum, and speaks accusingly not only of Dr. Bompas, but also of Dr. Prichard of Park Row, who was apparently responsible for her certification. She may well have been a case of anorexia nervosa, since one of her chief complaints was that she was forced to eat against her will too much food, "and that of the coarsest kind".

Like his great grandfather Joseph Mason, George Gwinnett Bompas was an ardent Baptist. Together with fourteen others, he broke away from Foster Baptist Church, Downend, in 1841 to form a separate body of worshippers in Fishponds. They held their first meeting in the asylum chapel, and presumably continued to meet there until Fishponds Baptist Church was opened in 1847, shortly after his death.

George Gwinnett Bompas died suddenly in February 1847—of angina pectoris, according to his obituary notice in the London and Provincial Medical Directory, which goes on to say that "he was of mild and amiable deportment, a model of the Christian gentleman, and all his actions were influenced by a feeling of deep responsibility. . . . Although not an implicit follower of what is called the non-restraint system, his judicious and gentle management of the patients entrusted to his care effected the successful restoration of the mental powers in a very remarkable degree."

After his death the asylum, which now housed nearly 50 patients, was taken over by his third surviving son, Dr. Joseph Carpenter Bompas, who was then only twenty-four years of age. He was very well qualified, having studied at University College, London, and taken a first class B.A. and a B.M. with honours in medicine and physiology. However, life was not to run smoothly for the youthful Dr. Bompas. By the end of 1847 the visiting magistrates were regularly making adverse reports, and between the 22nd November and the 2nd December 1848 an official enquiry was held at Lawford's Gate Sessions Room, on the instructions of the Gloucester Quarter Sessions. The report, entitled "The Evidence Taken on the Inquiry into the Management of the Fishponds Private Lunatic Asylum", is in the Bristol University Medical Library, and contains nearly 800 pages, mostly of verbatim evidence, examination and cross-examination. Hunter and Macalpine (1963) call it "the only printed public enquiry of such magnitude into the affairs of a single private madhouse". Some of the accusations seem to have been comparatively trivial—e.g. minor irregularities in keeping the statutory records; and others seem to have been based on the unsubstantiated word of patients. However, there is no doubt that what counted most against Dr. Bompas was the fact that he had allowed a particularly refractory patient to be restrained by a chain and an iron ring around his leg. In this he was evidently merely continuing to use the methods adopted by his father, and he had not had very long in which to introduce reform. On the other hand, we must remember that it was during the previous decade that the great move-

Plate IX. Part of the first Asylum 1740 - 1760

Plate X. The second Asylum 1760 - 1859

ment for the abolition of mechanical restraint had swept the country, led by Charlesworth and Gardiner Hill at Lincoln and by Conolly at Hanwell. Conolly himself gave evidence at the enquiry.

The Quarter Sessions made it clear that they would not renew his licence except for a temporary period, and he resigned in 1849. The asylum continued under other members of the Bompas family until 1852, and thereafter until 1859 under Dr. J. D. F. Parsons, who already had an asylum at Whitehall, Bristol.

During the next twenty years the premises were used first as a boot and shoe factory by Mr. Henry Massingham and later as a boys' school under the Reverend A. G. Morris. They were demolished about 1880 to make room for the houses which now occupy the site.

I am seeking further information about the places and people concerned and would greatly appreciate any help which readers of this paper may be able to give.

I am indebted to the following for their help: to Mr. W. M. G. Bompas of Cambridge for permission to reproduce the portrait of Joseph Mason; to Mr. J. A. Burgess, Managing Director of Hygienex Industries Ltd., for permission to photograph part of the firm's premises; to Mr. C. R. Hudleston of Durham for lending me his papers collected in 1938; to Mr. C. H. Massingham of Coventry for permission to reproduce the picture of the asylum; and to Mr. Leonard Nott of Fishponds for information about the founding of Fishponds Baptist Church.

### REFERENCES

Edinburgh Medical and Surgical Journal (1805). Vol. 1, p. 228.

**Evans, J.** (1824). A Chronological Outline of the History of Bristol, and the Stranger's Guide through its Streets and Neighbourhood. p. 269.

**Hunter, R., and Macalpine, I.** (1963). Three Hundred Years of Psychiatry 1535-1860, p. 594.

**Robinson, A. B., and Hudleston, C. R.** (1938). Two Vanished Fishponds Houses. Transactions of the Bristol and Gloucestershire Archaeological Society, Vol. LX, p. 238.

**Smith, Richard, Junior** (c. 1820). Bristol Infirmary Biographical Memoirs. Mss. in the Board Room, Bristol Royal Infirmary, pp. 160-171.

**Tuke, D. H.** (1882). Chapters in the History of the Insane in the British Isles, p. 513.

## Figures and Tables

**Plate VIII. f1:**
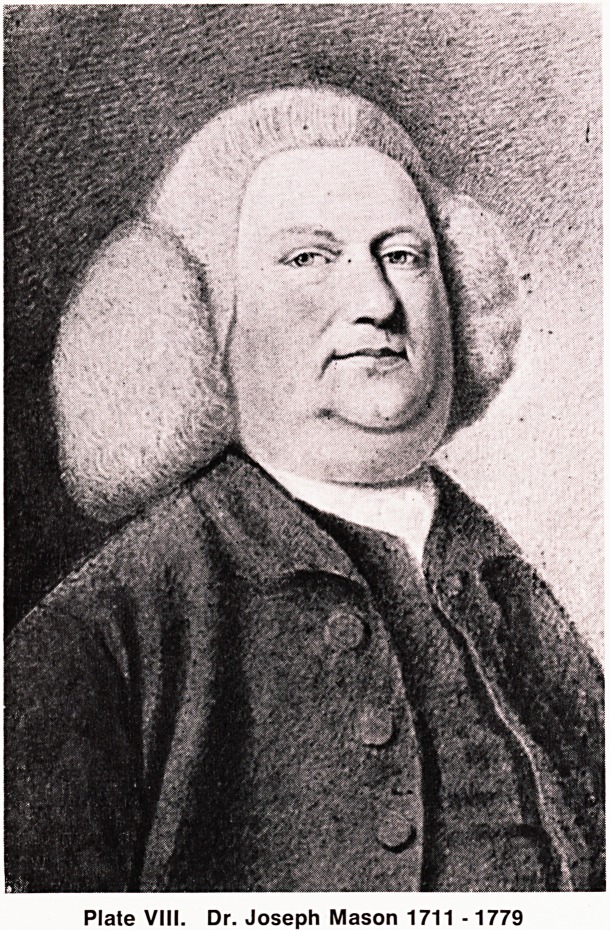


**Plate IX. f2:**
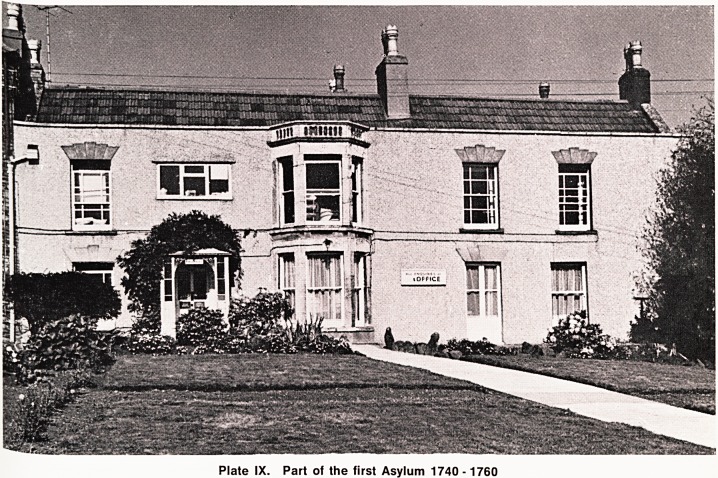


**Plate X. f3:**